# Augmentation of Nitric Oxide Deficient Hypertension by High Salt Diet Is Associated With Reduced TNF-α Receptor Type 1 Expression in the Kidneys

**DOI:** 10.1093/ajh/hpae066

**Published:** 2024-05-23

**Authors:** Dewan S A Majid, Minolfa C Prieto, Alexander Castillo, Cameron Chamberlain, Luis Gabriel Navar

**Affiliations:** Department of Physiology, Tulane Hypertension & Renal Center of Excellence, Tulane University School of Medicine, New Orleans, Louisiana 70112, USA; Department of Physiology, Tulane Hypertension & Renal Center of Excellence, Tulane University School of Medicine, New Orleans, Louisiana 70112, USA; Department of Physiology, Tulane Hypertension & Renal Center of Excellence, Tulane University School of Medicine, New Orleans, Louisiana 70112, USA; Department of Physiology, Tulane Hypertension & Renal Center of Excellence, Tulane University School of Medicine, New Orleans, Louisiana 70112, USA; Department of Physiology, Tulane Hypertension & Renal Center of Excellence, Tulane University School of Medicine, New Orleans, Louisiana 70112, USA

**Keywords:** blood pressure, hypertension, natriuresis, renal tissue, salt-sensitive hypertension, TNF-α, TNF-α receptor type 1 (TNFR1)

## Abstract

**BACKGROUND:**

High salt (HS) intake induces an augmented hypertensive response to nitric oxide (NO) inhibition, though it causes minimal changes in blood pressure (BP) in NO intact condition. The cause of such augmentation is not known. HS induces tumor necrosis factor-alpha (TNFα) production that causes natriuresis via activation of its receptor type 1 (TNFR1). We hypothesized that NO deficiency reduces renal TNFR1 activity, leading to enhanced sodium retention and hypertension.

**METHODS:**

We examined the changes in renal TNFR1 protein expression (Immunohistochemistry analyses) after HS (4% NaCl) intake in wild-type mice (WT, C57BL6) treated with a NO synthase (NOS) inhibitor, nitro-l-arginine methyl ester (L-NAME; 0.05 mg/min/g; osmotic mini-pump), as well as in endothelial NOS knockout mice (eNOSKO) and compared the responses in WT mice with normal salt (NS; 0.3% NaCl) intake. BP was measured with tail-cuff plethysmography and 24-hour urine collections were made using metabolic cages.

**RESULTS:**

HS alone did not alter mean BP in untreated mice (76 ± 3 to 77 ± 1 mm Hg) but induced an augmented response in L-NAME treated (106 ± 1 vs. 97 ± 2 mm Hg) and in eNOSKO (107 ± 2 vs. 89 ± 3 mm Hg) mice. The percentage area of TNFR1 expression in renal tissue was higher in WT + HS (4.1 + 0.5%) than in WT + NS mice (2.7 ± 0.6%). However, TNFR1 expression was significantly lower in L-NAME treated WT + NS (0.9 ± 0.1%) and in eNOSKO + NS (1.4 ± 0.2%) than in both WT + NS and WT + HS mice.

**CONCLUSIONS:**

These data indicate that TNFR1 activity is downregulated in NO deficient conditions, which facilitates salt retention leading to augmented hypertension during HS intake.

## Graphical Abstract

**Graphical Abstract:**
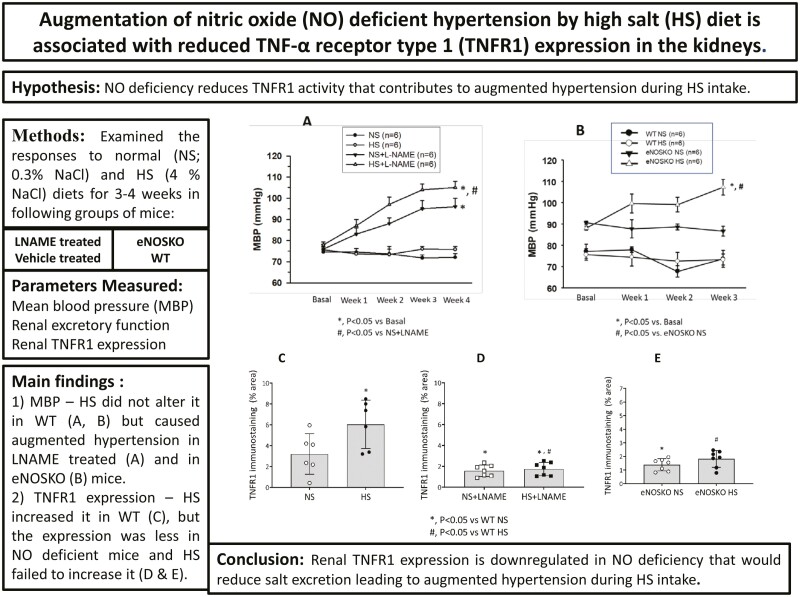

Salt-sensitive hypertension (SSH) in humans is characterized by a sustained increase in arterial pressure induced by high salt (HS) diets. Chronic HS intake, through an osmoreceptor function, induces an immune mechanism that activates mononuclear phagocyte system (MPS) cells in the myeloid tissues.^1–5^ These activated MPS cells release proinflammatory cytokines, particularly tumor necrosis factor-alpha (TNFα), which is implicated in the pathogenesis of SSH and renal injury.^1–5^ TNFα levels in the kidneys are also increased in hypertensive conditions induced by angiotensin II (AngII),^6,7^ nitric oxide (NO) deficiency^8–10^ as well as in Dahl salt-sensitive rats fed a HS diet.^11^

TNFα exerts renal responses by activating its’ two receptors: type 1 (TNFR1) and type 2 (TNFR2). TNFR1 receptors are located in cells of renal proximal tubules, collecting duct, vascular endothelium, and in vascular smooth muscle, whereas TNFR2 receptors are located in cells of renal proximal tubule, collecting duct, and vascular endothelium, but not in the vascular smooth muscle cells.^12,13^ TNFR1 activation results in increased urinary excretion of sodium (U_Na_V),^8,13^ indicating that this receptor’s function provides a counter-acting mechanism in hypertension by opposing salt retention.^14^ However, the activation of TNFR2 is involved in inflammation-induced macrophage infiltration and renal injury induced by AngII.^15^

Although HS intake alone induces minimal changes in blood pressure (BP), it augments the hypertensive and renal injury responses to chronic NO inhibition.^16–18^ The mechanism for such responses to HS intake during NO inhibition is not yet clearly understood.^18^ TNFα level is increased in plasma but its’ level is decreased in renal tissues during chronic HS intake in NO deficient conditions.^10,14^ Thus, it is possible that sodium excretory function altered by TNFR1 activation in the kidneys is compromised in NO deficient conditions which would play a role in the increased hypertensive response.

The present study is designed to examine the hypothesis that the TNFR1 activity is downregulated in the kidney during NO inhibition, which minimizes sodium excretion, leading to enhanced salt retention resulting in an augmented hypertensive response during HS intake. Here, we have assessed the renal responses to HS intake in NO-deficient mice with particular emphasis on the changes in protein expression of TNFR1 and TNFR2 in the renal tissues.

## METHODS

All experimental procedures were approved by and performed following the guidelines and practices set up by the Tulane University Animal Care and Use Committee. Knockout (KO) mice lacking the gene for endothelial nitric oxide synthase (NOS) (eNOSKO; B6.129P2-Nos3tm1Unc/J; stock no: 002684) and their control genetic background strain of Wild type mice (WT; C57BL/6J; stock no: 002684), purchased from Jackson Laboratory (Bar Harbor, ME), were used in this study. Male mice were used mainly to avoid estrous cycle-dependent data variability and to obtain data comparable to the previous experiments using similar species and genders. Mice were housed in a temperature- and light-controlled room on a 12:12-hour light-dark cycle and received food and water *ad libitum* throughout the study. The mice were 8–9 weeks of age and of ∼25 g body weight, bw. These were divided into separate groups depending on the intake of the NS diet (a standard diet having 0.3% NaCl; Ralston-Purina, St. Louis, MO) and HS diet (HS, 4% NaCl; Harlan-Teklad, Madison, WI), respectively.

Two models of NO deficient mice are used in this study: (i) WT mice chronically treated with L-NAME and (ii) eNOSKO mice. L-NAME was administered in WT mice for 4 weeks by implanted mini-pump at a rate of 0.05 mg/min/g. The mice were given either NS or HS diets and divided into the following experimental groups:

1) L-NAME treated groups
  a) L-NAME + NS (*n* = 7)
  b) L-NAME + HS (*n* = 7)
  c) Vehicle + NS (*n* = 6)
  d) Vehicle + HS (*n* = 6)
2) eNOSKO groups
  a) eNOSKO + NS (*n* = 7)
  b) eNOSKO + HS (*n* = 7)
  c) WT + NS (*n* = 6)
  d) WT + HS (*n* = 6)

The experimental period was 1 week more in L-NAME treated groups (4 weeks) than in eNOSKO groups (3 weeks) to allow a stabilization period following installation of osmotic mini-pumps for L-NAME administration.

### Measurement of BP

The systemic BP was measured using the non-invasive tail-cuff plethysmography technique (Visitech Systems, Apex, NC) which allows recordings of systolic, diastolic, and mean BP (MBP) using its Analysis Software. BP was measured in mid-daytime at the start (Day 0) and at the end of every week of the experimental periods by estimating the average reading of 10 measurements for a single trial. The mice were trained for tail-cuff BP measurements 3 days before starting the experiments. The basal BP was measured in all the mice on the NS diet before the start of the HS diet in the designated groups.

### Urine collection

24-hour urine samples were collected into sterile tubes from mice housed individually in metabolic cages on Day 0 (basal excretory parameters), and then at the end of every week of experimental periods. Maximum precaution was taken to avoid contamination of urine with chow food debris by covering the collection tubes with cling-film. Urine volumes were determined, samples were centrifuged (3,000 rpm/5 min; 4 °C), and the concentrations of sodium and potassium were assessed by flame photometry. At the end of the experiments, the mice were sacrificed, and the kidneys were isolated and processed for tissue analysis.

### TNFR1 and TNFR2 protein expression in renal tissue

Decapsulated kidneys were embedded in paraffin blocks, sectioned (3 μm), and mounted onto slides with Vectabond (Vector Laboratories, Burlingame, CA). Serial kidney sections having tissues from both the renal cortex and medulla, were immunoassayed using the immunoperoxidase technique for analyzing renal tissue TNFR1 and TNFR2 expression that was used in our earlier studies.^13,19,20^ Briefly, the rehydrated renal sections were sequentially incubated with (i) normal blocking rabbit serum for 30 min, (ii) primary antibodies [rabbit polyclonal anti-TNF receptor 1 antibody (catalog no. ab19139, Abcam, Cambridge, MA) or rabbit monoclonal anti-TNF receptor 2 antibody (catalog no. 3605-1, Epitomics, Burlingame, CA)] diluted in normal blocking serum (Vector Laboratories) at 1:1,000 and 1:200 for TNFR1 and TNFR2, respectively, overnight at 4 °C, (iii) secondary antibody [biotin-conjugated rabbit anti-mouse IgG (Vector Laboratories)] for 30 min, and (iv) avidin DH (biotinylated horseradish peroxidase H complex) using the ABC Elite Vectastain kit (Vector Laboratories) for 30 min. The slides were counterstained with hematoxylin (VWR International, West Chester, PA). Specific immunoreactivity was quantified in digital images captured from 20 different microscopic fields per tissue section per animal using a Nikon Eclipse 50i microscope, a ×40 objective, and an integrated digital camera system for image processing. The intensity of TNFR1 and TNFR2 immunoreactivity in all sections was analyzed using NIS-Elements Software AR (version 3.0 for Windows; Nikon), which allowed a computerized determination of the area of positive staining (μm) and the intensity of immunoreactivity (sum of density in an analyzed area). The results are expressed in arbitrary units of the relative intensity and normalized to the mean values of specific immunostaining observed in the control mice. TNFR1 and TNFR2 are expressed both in renal proximal and distal tubular collecting duct cells as well as in renal vasculature,^13^ we measured the total immunoreactivity in all sections having tissues from both the renal cortex and medulla.

### Estimation of glomerulosclerosis

This was evaluated quantitatively by automatic image analysis of each glomerulus using Periodic-Acid-Schiff (PAS)-stained renal sections (Mass Histology Service, Worcester, MA).^21,22^ Twenty images from each kidney slide with at least one glomerulus per field were photographed using a Nikon Eclipse 50i microscope equipped with a Nikon DS Camera Head (DS Fi1) and DS camera control unit (DSU2). A dark purple color in the glomerulus was recognized as sclerosis. The percentage area covered by sclerosis in glomeruli in each field was analyzed using the Nikon NIS-Elements software (version 2.34) and results from 20 images were averaged to obtain the percentage area of sclerosis for the entire slide.

### Estimation of renal interstitial fibrosis

This was evaluated quantitatively by automatic image analysis of the renal sections occupied by interstitial tissue staining positively for collagen in Gomori’s trichrome staining.^23^ Formalin-fixed paraffin-embedded sections were stained with a plasmin stain (chromotrope 2R) and a connective tissue fiber stain (aniline blue) combined in a solution of phosphotungstic acid to which glacial acetic acid had been added. This stained the collagen in blue, which indicates fibrosis. Slides were photographed as described above. The percentage area covered by collagen in each field was analyzed using the Nikon NIS-Elements software (version 2.34) and data obtained from 20 images were averaged to obtain the percentage area of fibrosis for the entire slide.

### Statistical analysis

Results were expressed as mean ± SE. All excretory values were normalized as units per gram of kidney weight. Statistical analysis was performed using SigmaStat software (Systat Software, Chicago, IL). The values within groups were compared using the repeated measure analysis of variance (ANOVA) and Dunnett multiple comparisons test. Student’s *t*-tests were used for comparison of the responses between the groups. Differences are considered statistically significant at *P* values < 0.05.

## RESULTS

**Figure 1.**
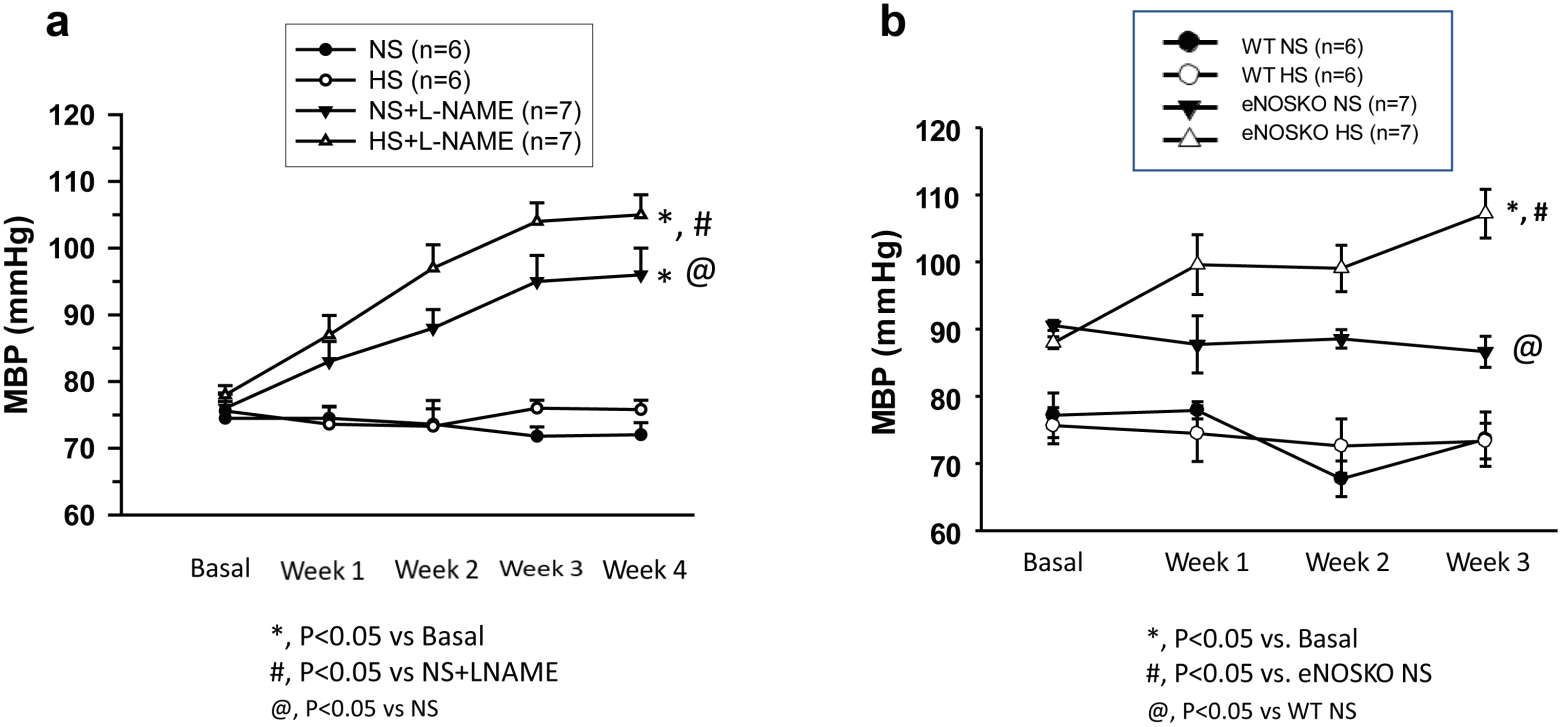
Mean blood pressure (MBP) responses to normal (NS) and high salt (HS) intake in chronically treated mice with nitro-l-arginine methyl ester (L-NAME) (**a**) and in eNOSKO mice (**b**).

1) BP responses
  a) L-NAME groups: Figure 1a illustrates the changes in MBP during chronic administration of L-NAME/vehicle during NS and HS intake. HS intake alone did not alter MBP (76 ± 3 to 77 ± 1 mm Hg; *n* = 6; *P* = n.s.) during the experimental period. L-NAME treatment for 4 weeks in the NS group caused increases in MBP (74 ± 4 to 97 ± 2 mm Hg; *P* < 0.05; *n* = 7). Although HS alone did not alter MBP, it augmented the L-NAME-induced increases in MBP (from 97 ± 2 to 106 ± 1 mm Hg; *P* < 0.05; *n* = 7).
  b) eNOSKO groups: As illustrated in Figure 1b, HS intake in WT mice did not alter MBP (74 ± 2 to 75 ± 3 mm Hg; *n* = 6; *P* = n.s.) during 3 weeks of the experimental period. The baseline BP was higher in eNOSKO (91 ± 2 vs. 75 ± 2 mm Hg; *P* < 0.05) than in WT mice. HS intake in eNOSKO mice increased MBP (107 ± 2 mm Hg; *P* < 0.05) but not during NS intake (89 ± 3 mm Hg). These results were comparable to the findings in our previous study with WT and eNOSKO mice earlier^10^ where we measured BP every 3 days for 2 weeks during chronic intake of NS and HS diets.
2) Changes in urinary parameters

Both the absolute values and the percentage changes in renal excretory parameters (urine flow, urinary sodium excretion, and urinary potassium excretion) are shown in Table 1. As shown, the increases in urine flow and sodium excretion during HS intake were less in NO-deficient mice than in vehicle-treated WT mice.

**Table 1.** Changes in renal excretory function in NO deficient mice during varying salt (NS/HS) intake

Parameter	NS—WT		HS—WT		NS—eNOSKO		HS—eNOSKO		NS—L-NAME		HS—L-NAME	
	Basal	End of expt. period	Basal	End of expt. period	Basal	End of expt. period	Basal	End of expt. period	Basal	End of expt. period	Basal	End of expt. period
Urine flow												
Absolute values (mL/day)	1.1 ± 0.2	0.9 ± 0.1, ns	0.9 ± 0.1	2.8 ± 0.3*	1.1 ± 0.1	1.2 ± 0.1, ns	1.4 ± 0.2	3.2 ± 0.3*	1.1 ± 0.2	0.81 ± 0.1, ns	0.92 ± 0.1	1.6 ± 0.3*
Percent changes (%)		−3.5 ± 20, ns		230 ± 45 *^,^^#^		10 ± 16, ns		123 ± 21*,^#^		−12 ± 21, ns		84 ± 25*^,^^#^
Urinary sodium excretion												
Absolute values (µM/day)	129 ± 13	133 ± 12, ns	138 ± 16	1,254 ± 102*	135 ± 13	125 ± 12, ns	156 ± 15	1,037 ± 101*	129 ± 14	134 ± 10, ns	122 ± 13	773 ± 142
Percent changes (%)		7.4 ± 13, ns		866 ± 123*^,^^#^		−1.2 ± 13, ns		574 ± 54*^,^^#^		14 ± 19, ns		538 ± 126*^,^^#^
Urinary potassium excretion												
Absolute values (µM/day)	272 ± 51	275 ± 32, ns	264 ± 33	381 ± 27, ns	278 ± 26	279 ± 45, ns	303 ± 30	365 ± 29, ns	251 ± 40	261 ± 28, ns	257 ± 32	274 ± 31, ns
Percent changes (%)		19 ± 28, ns		57 ± 22, ns		3 ± 14, ns		25 ± 13, ns		26 ± 31, ns		14 ± 18, ns

ns, not significant vs. Basal values.

Abbreviations: HS, high salt; L-NAME, nitro-l-arginine methyl ester; NO, nitric oxide; NS, normal salt; WT, wild-type.

*P < 0.05 vs. corresponding Basal values;

^#^ *P* < 0.05 vs. changes in WT-HS group.

**Figure 2.**
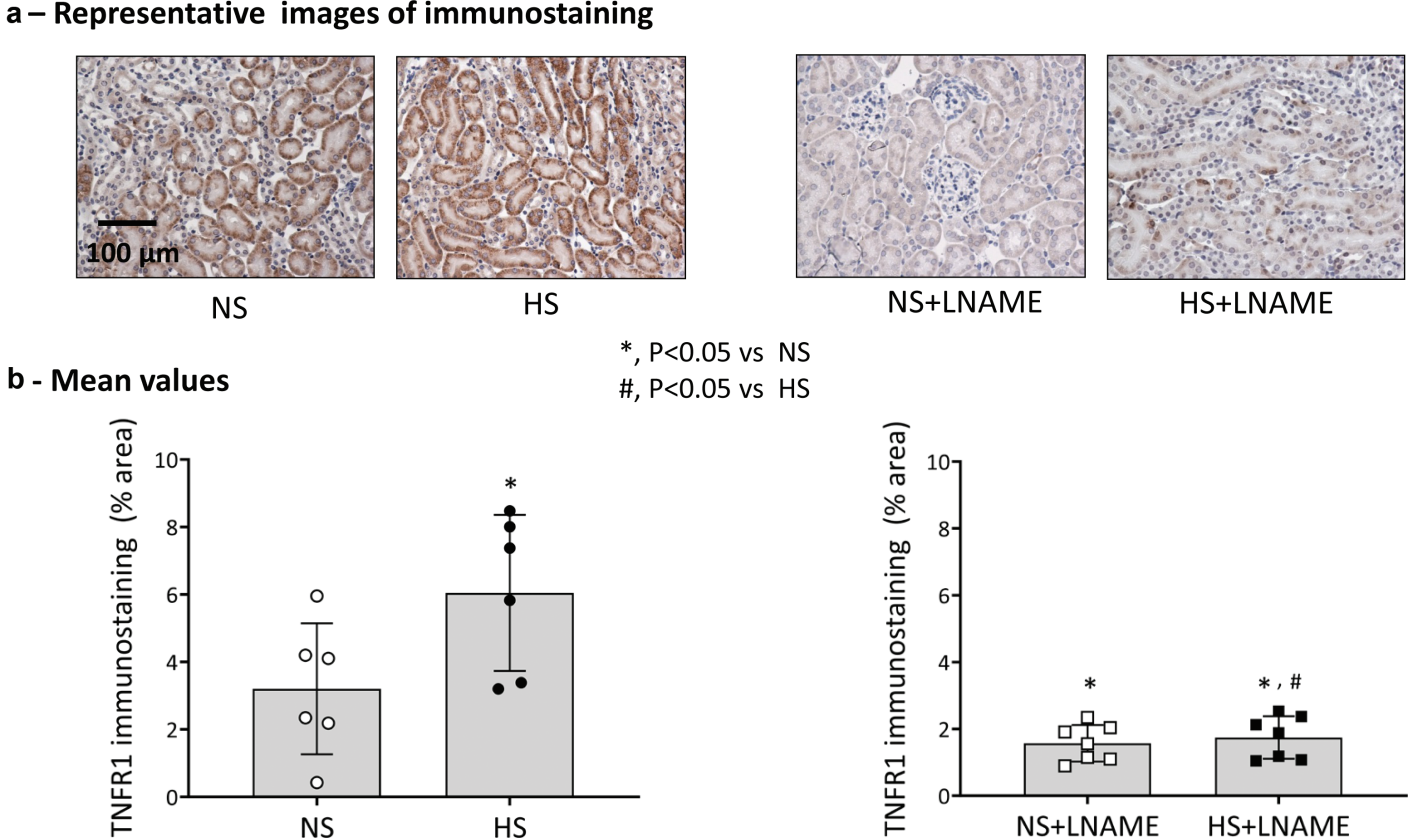
Renal tissue tumor necrosis factor receptor type 1 (TNFR1) immunostaining responses to normal (NS) and high salt (HS) intake in mice chronically treated with nitro-l-arginine methyl ester (L-NAME). (**a**) Shows representative images and (**b**) provides the values (percentage area of tissues) of the images. The scale bar indicates 100 µm.

**Figure 3.**
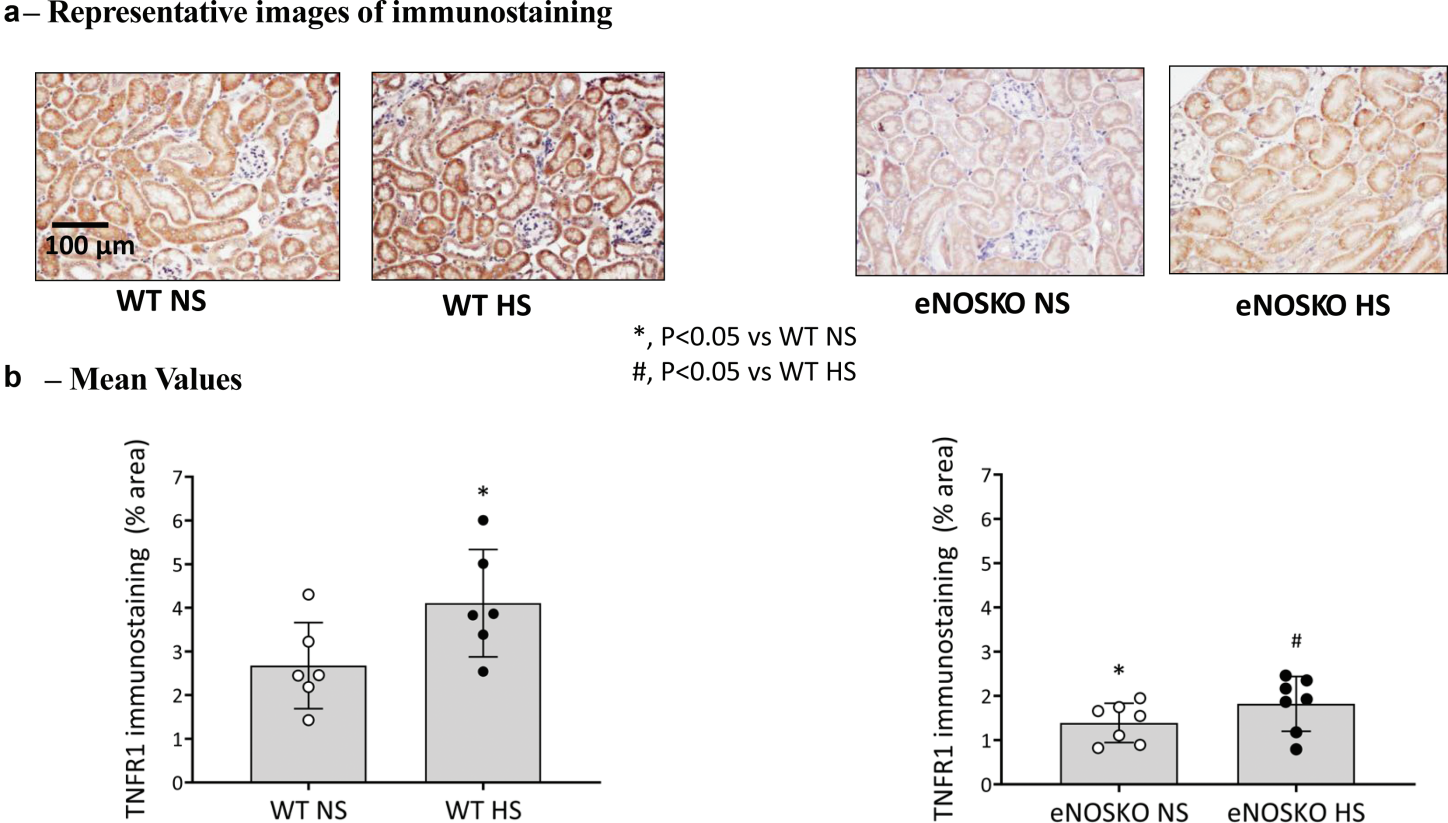
Renal tissue tumor necrosis factor receptor type 1 (TNFR1) immunostaining responses to normal (NS) and high salt (HS) intake in eNOSKO mice. (**a**) Shows the representative images and (**b**) provides values (percentage area of tissues) of the images. The scale bar indicates 100 µm.

3) TNFR1 protein expression in renal tissue
  a) L-NAME groups: Figure 2 illustrates the changes in TNFR1 immunostaining in renal tissues collected. Figure 2a illustrates the representative images and Figure 2b gives the mean values (percentage area of tissues) of immunostainings in different groups. Renal tissue TNFR1 protein expression was higher in the HS intake group than in the NS group. However, this expression was suppressed in L-NAME treated groups fed NS and HS and were not different from each other.
  b) eNOSKO groups: Figure 3 illustrates the changes in renal tissue TNFR1 expression in eNOSKO groups. Figure 3a shows the representative images and Figure 3b gives the mean values (percentage area of tissues) of immunostaining in different groups. TNFR1 protein expression was significantly lower in eNOSKO than in WT mice and HS intake did not increase TNFR1 expression in eNOSKO mice.
4) TNFR2 protein expression in renal tissue

**Figure 4.**
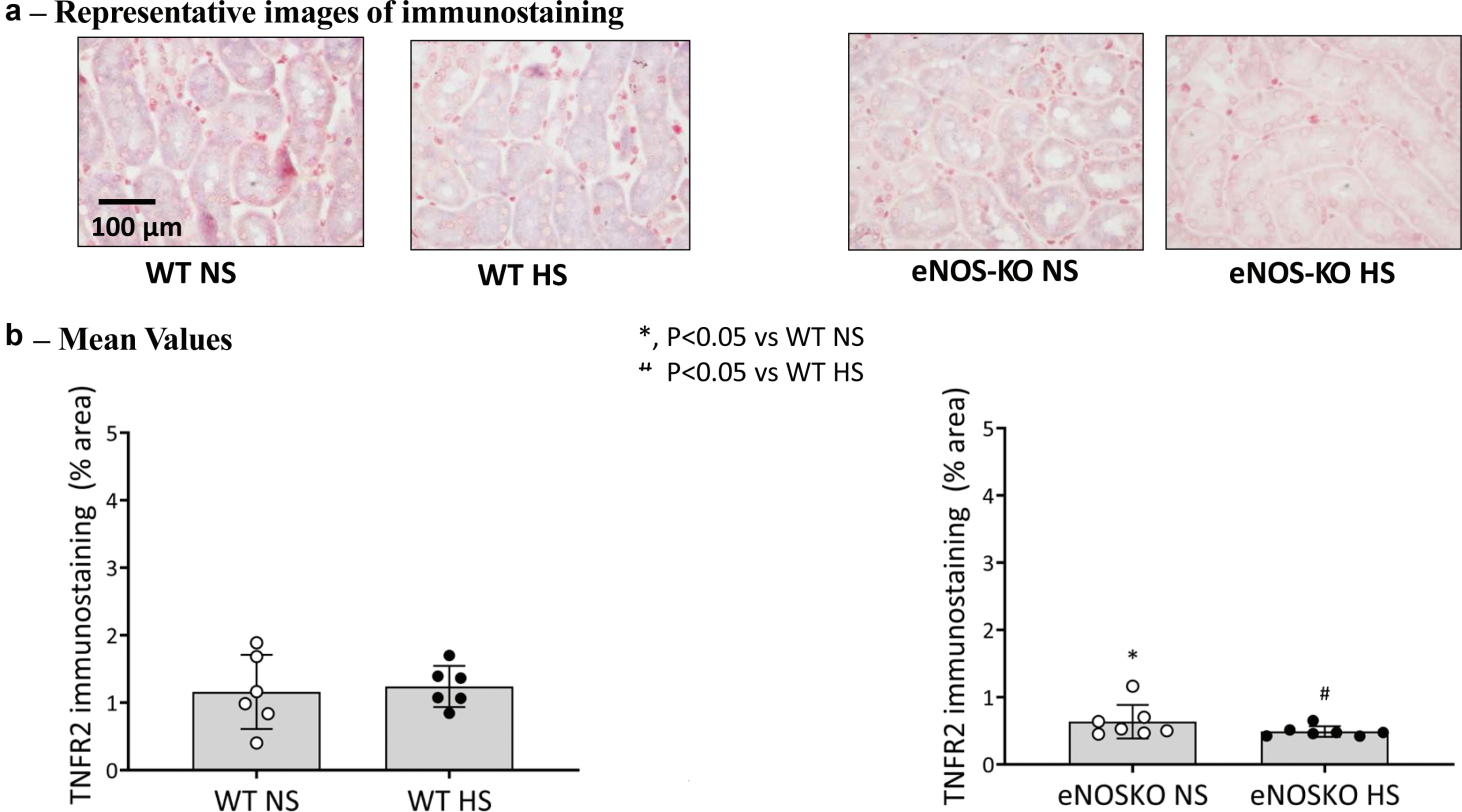
Renal tissue tumor necrosis factor receptor type 2 (TNFR2) immunostaining responses to normal (NS) and high salt (HS) intake in eNOSKO mice. (**a**) Shows the representative images and (**b**) provides values (percentage area of tissues) of the images. The scale bar indicates 100 µm.

Figure 4 illustrates the changes in renal tissue TNFR2 expressions in eNOSKO groups. Figure 4a provides representative images and Figure 4b gives the mean values (percentage area of tissues) of immunostainings in different groups. The changes in TNFR2 expression in L-NAME treated as well as in vehicle-treated mice are not given as these are comparable to that in eNOSKO mice and in WT mice. TNFR2 protein expression was relatively low (~1.5% staining area) and not different in NS and HS groups. However, this expression was further suppressed in both NO deficient mice (L-NAME treated or eNOSKO) fed either NS or HS diet.

5) Renal injury responses: The extent of glomerular sclerosis as determined by PAS-staining^21^ and the extent of interstitial fibrosis as determined using Gomori’s trichrome staining are shown in Table 2 which provides the mean glomerulosclerotic (GS) and collagen depositions scores (% area of staining) from different groups of mice. GS scores were low in WT mice in both NS intake and HS intake groups. However, the GS scores were significantly higher in NO deficient mice (both L-NAME treated and eNOSKO) fed with NS and HS diets. HS intake alone did not alter collagen deposition in WT mice, but these were significantly higher in both eNOSKO and L-NAME treated mice.

**Table 2.** Renal injury scores in NO deficient mice during varying salt (NS/HS) intake

	NS—WT	HS—WT	NS—eNOSKO	HS—eNOSKO	NS—vehicle	HS—vehicle	NS—L-NAME	HS—L-NAME
Glomerulosclerotic scores (PAS staining) (% area of staining)	0.9 ± 0.2	1.0 ± 0.1, ns	5.2 ± 0.4*	6.5 ± 0.6^#^	0.7 ± 0.2	0.8 ± 0.3, ns	5.0 ± 0.3*	7.5 ± 0.4^#^
Renal interstitial fibrosis scores (Gomori’s trichrome staining) (% area of staining)	0.3 ± 0.1	0.6 ± 0.3, ns	2.8 ± 0.7*	3.7 ± 0.4^#^	0.4 ± 0.2	0.5 ± 0.3, ns	1.9 ± 0.3*	2.3 ± 0.4^#^

ns, not significant vs. NS-WT or NS-Vehicle values.

Abbreviations: HS, high salt; L-NAME, nitro-l-arginine methyl ester; NO, nitric oxide; NS, normal salt; PAS, Periodic-Acid-Schiff; WT, wild-type.

*P < 0.05 vs. NS-WT or NS-Vehicle values;

^#^ *P* < 0.05 vs. HS-WT or HS-Vehicle.

## DISCUSSION

The results of the present study demonstrate that the expression of TNFR1 protein in the renal tissue is downregulated in NO-deficient mice. Although chronic HS intake enhances the renal TNFR1 expression in NO intact mice, this response is absent in NO deficient mice (Figures 2 and 3). As reported in our previous studies,^10,17^ the present study also shows that chronic HS intake caused minimal changes in BP in intact mice but induced an augmented hypertensive response in NO-deficient mice (Figure 1). An augmented hypertensive response to chronic AngII administration during HS intake in mice was previously shown to be associated with reduced expression of TNFR1 in the kidney.^20^ Previous studies also demonstrated that chronic HS augmented the hypertensive response to AngII administration in mice lacking the gene for TNFR1.^21,23^

Chronic HS intake usually induces an immune mechanism that activates MPS cells in the myeloid tissues in the bone marrow, spleen, and skin tissue^3,5,24^ and these activated MPS cells release TNFα that enters the circulation.^4,6,10^ The increased circulating TNFα induces natriuresis via its’ action on TNFR1 in the kidney^8,13,25^ reflecting a protective role for TNFR1 during HS intake.^21^ Usually, a minimal change in BP during HS intake occurs mainly due to consequent increases in NO production,^26,27^ which increase sodium excretion^25^ and thus, maintain normal sodium balance leading to minimal changes in extra-cellular fluid (ECF) volume.^18^ The results of the present investigation also reveal that the protective role of TNFR1 is an additional mechanism for maintaining normal ECF volume and BP during chronic HS intake.^14,24^ As the present findings demonstrate, this protective role for TNFR1 during HS intake is compromised under NO deficient conditions, thus causing salt retention leading to augmented hypertensive responses to chronic HS intake.

A mechanistic explanation for the upregulation of TNFR1 protein expression in NO intact condition and its’ downregulation in NO deficient conditions is not clear yet. In NO-deficient mice, the downregulation of TNFR1 could be related to the activation of NFkB due to oxidative stress that posed an apoptotic lesion to limit its’ expression as demonstrated in an *in vitro* study earlier.^28^ In the presence of NO, NFkB formation is minimal and thus, permits upregulation of TNFR1 protein expression as observed in the present study and consistent with previous study^29^ that NO donor treatment upregulates TNFRI mRNA gene expression in a dose-dependent manner in cultured endothelial cells derived from human umbilical veins suggesting a generalized response. As chronic HS intake induces increases in NO production,^10,22,30^ could explain the enhancement in TNFR1 expression in the renal tissue. It seems that NO production by eNOS isoforms is mainly involved in modulating TNFRI mRNA gene expression as TNFR1 expression during HS intake was similar in L-NAME treated as well as in eNOSKO mice in the present study. It has also been reported that chronic HS intake mainly involves eNOS isoforms to induce NO production in the vascular system^27^ as well as in the renal tubules.^26,31^

In the present study, HS intake alone did not induce renal injury (glomerulosclerosis and interstitial fibrosis) either in NO intact or in NO deficient mice. However, the injury score increased in NO deficient conditions though there was downregulation of both TNFR1 and TNFR2 expression. These findings show that the cytokine responses to chronic HS intake are not directly linked to renal injury induced by NO deficiency and oxidative stress. In a previous study,^10^ we reported that chronic HS intake for 2 weeks did not enhance renal injury scores, but rather slightly decreased them both in WT and in eNOSKO mice.

Although investigated for decades, uncertainty remains regarding how HS intake is mechanistically linked to the development of SSH in humans.^18,24^ Inability to explain how HS raises BP in some individuals (“salt-sensitive”) but not in others (“salt-resistant”) has hampered the development of a comprehensive therapeutic approach for SSH.^18^ The present findings showing the role of renal TNFR1 activity in NO-deficient and intact conditions provides a mechanistic explanation for the BP heterogeneity in response to HS intake in humans. These results show that HS intake increases renal TNFR1 activity, which increases salt excretion, thus minimizing BP increases and providing an explanation for the “salt-resistant” phenotype. In contrast, a decrease in renal TNFR1 activity in NO deficient conditions causes salt retention leading to an increase in BP recognized as a “salt-sensitive” phenotype.^18^ As NO deficiency is generally observed to be linked with salt sensitivity,^17,32^ this linkage provides the basis for targeting strategies to enhance TNFR1 activity as an approach to the prevention of SSH in humans. In contrast, as the present findings implied, it is reasonable to expect that treatment with inhibitors of TNFR1 activity such as the use of antibodies for TNFR1 would exaggerate SSH in patients. This is evident when it is reported from a meta-analysis of randomized control trials that anti-TNF therapy is associated with a significantly increased risk of developing hypertension in patients with rheumatoid arthritis.^33^ This trial renders a caution that the physicians should be aware of this risk and provide continuing monitoring in patients receiving these therapies. It may be mentioned here that a lack of commercially available specific TNFR1 antibody precludes us from confirming this risk experimentally during HS intake in animals. All the available TNF receptor antibodies are either non-specific or have a greater affinity to the TNFR2 receptor.^34^ There are currently five approved TNF biologics that work by completely blocking the interaction of TNFα with its receptors non-specifically, but to date, there are no small molecule therapeutics available that disrupt the high-affinity TNFα–TNFR1 interaction.^34^

In conclusion, these experimental findings show that NO deficiency downregulates the TNFR1 protein expression in the kidney. As TNFR1 plays an important protective role against sodium retention during HS intake, such downregulation of TNFR1 protein expression/activity in the condition of NO deficiency explains the enhancement in salt retention leading to an exaggerated hypertensive response to chronic HS intake.
